# Supplementary figures and images for: Comparison of the antibacterial properties of peptides from the hepatopancreas of red king crab and snow crab and development of an approach for red king crab peptide isolation from the hepatopancreas
Source: PeerJ. 2025 Sep 16;13:e19989. doi: 10.7717/peerj.19989 (PMC12447949; doi:10.7717/peerj.19989)

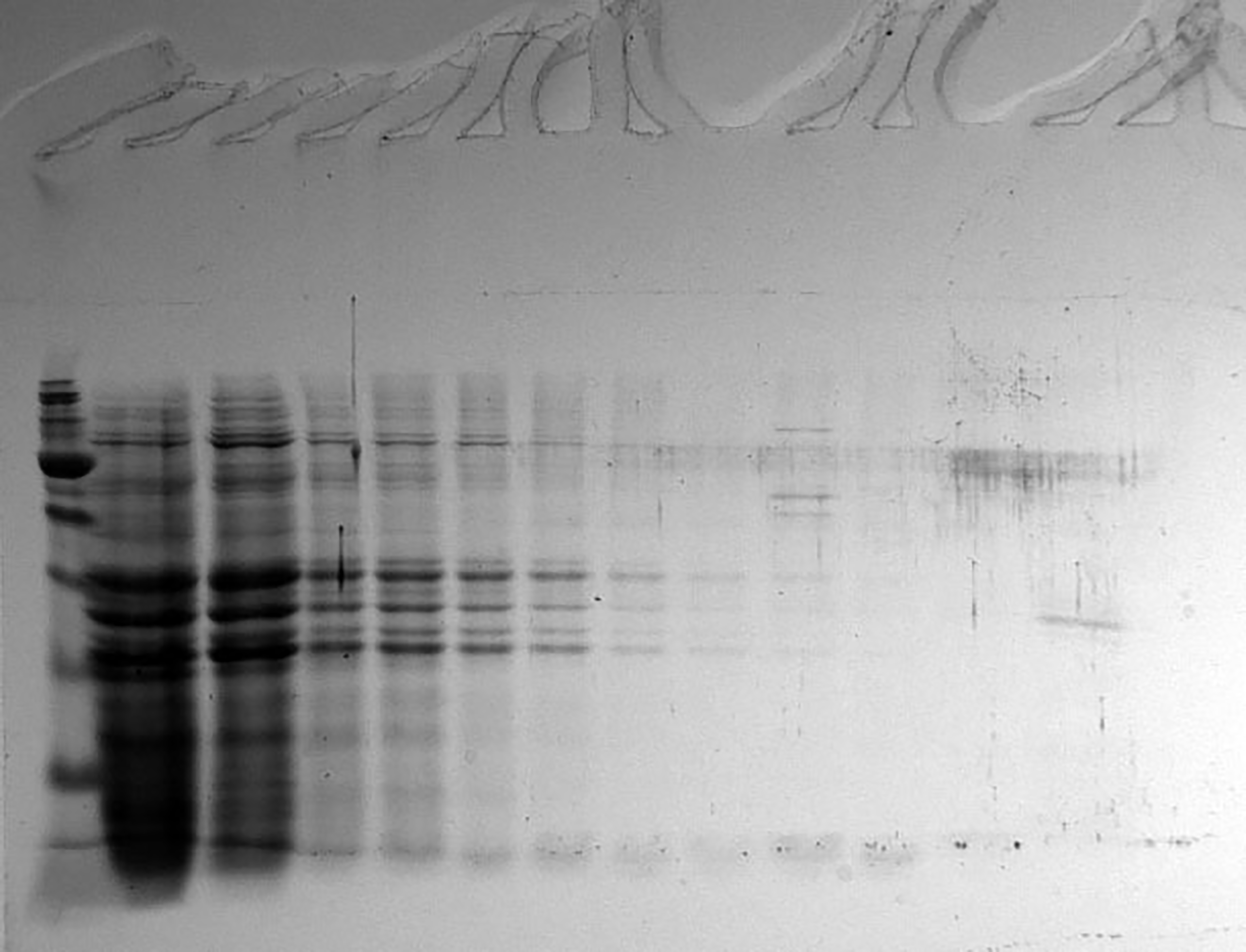

Supplement: Supplemental Information 1 — Lanes: M, molecular weight markers; 1, unbound proteins fractions from Blue Sepharose; 2–4. column washing; 5, fraction eluted in five mM NaCl; 6, fraction eluted in 10 mM NaCl; 7, fraction eluted in 20 mM NaCl; 8, fraction eluted in 30 mM NaCl; 9, fraction eluted in 40 mM NaCl; 10, fraction eluted in 50 mM NaCl; 11, fraction eluted in 75 mM NaCl; 12, fraction eluted in 100 mM NaCl; 13, fraction eluted in 500 mM NaCl. [file peerj-13-19989-s001.jpg]

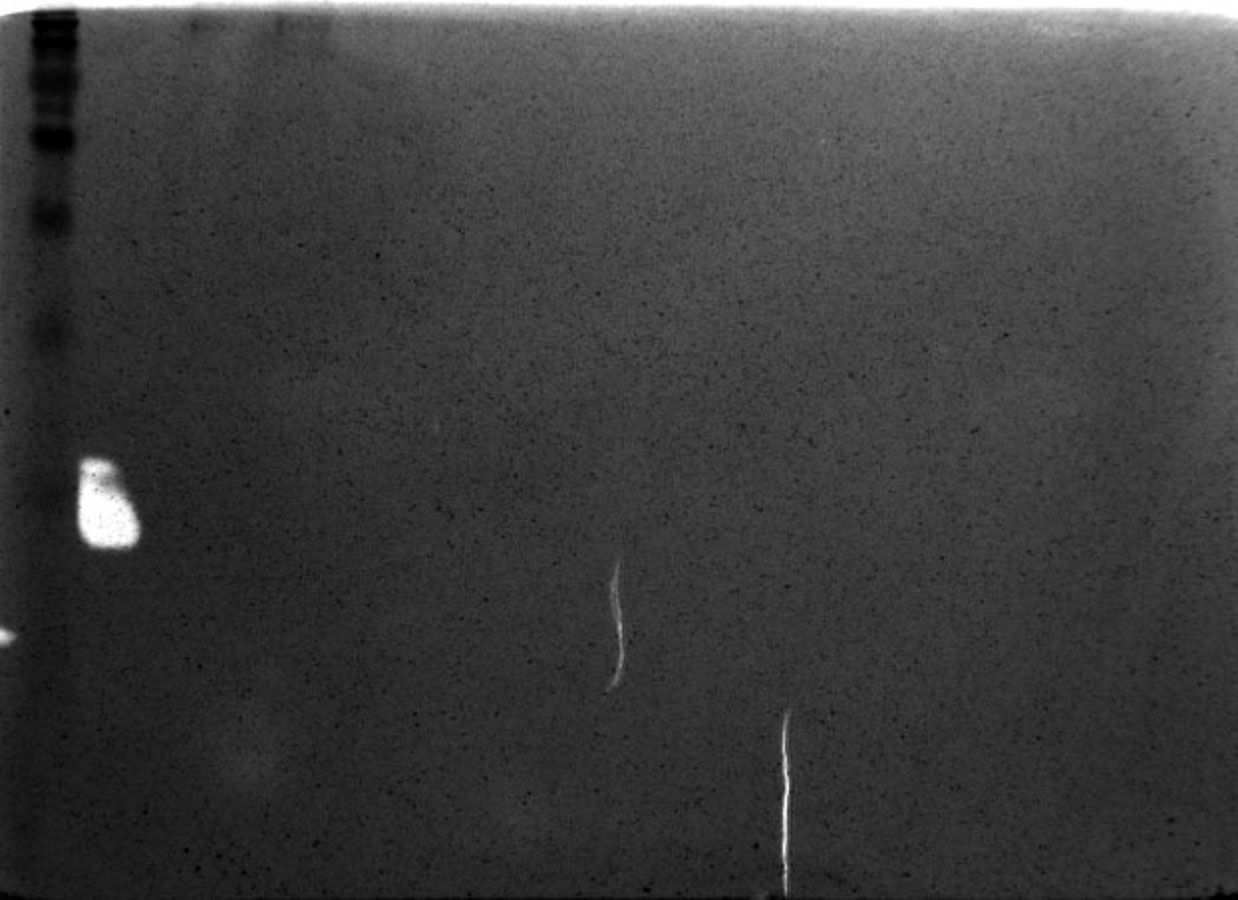

Supplement: Supplemental Information 2 — Lanes: M, molecular weight markers; 1, HEWL; 2, empty lane; 3, unbound proteins fraction from the Blue Sepharose; 4, empty lane; 5, fraction eluted in 10 mM NaCl from the Blue Sepharose. [file peerj-13-19989-s002.jpg]

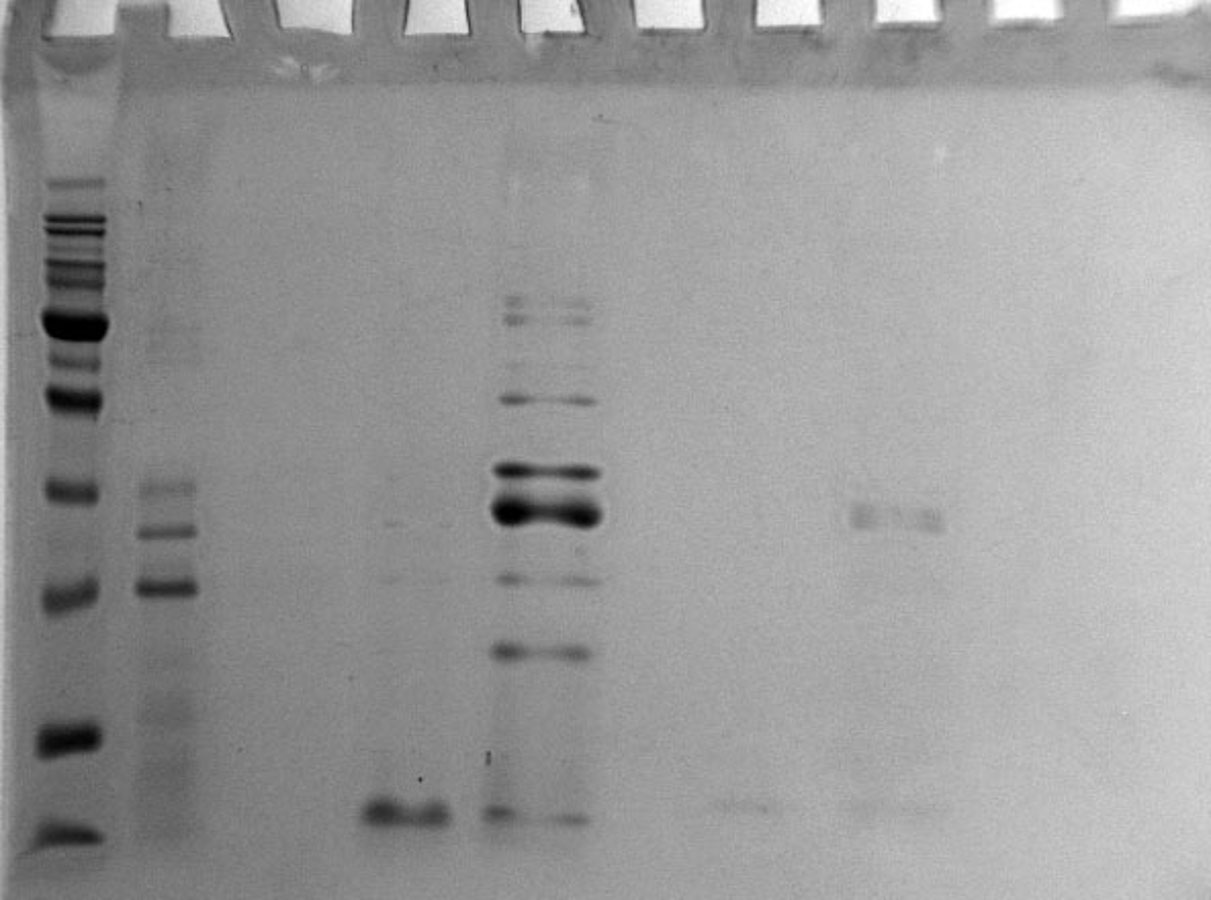

Supplement: Supplemental Information 3 — Lanes: M, molecular weight markers; 1, unbound protein fraction from Blue Sepharose 6 Fast Flow; 2, empty lane; 3, fraction eluted in 10 mM NaCl. [file peerj-13-19989-s003.jpg]

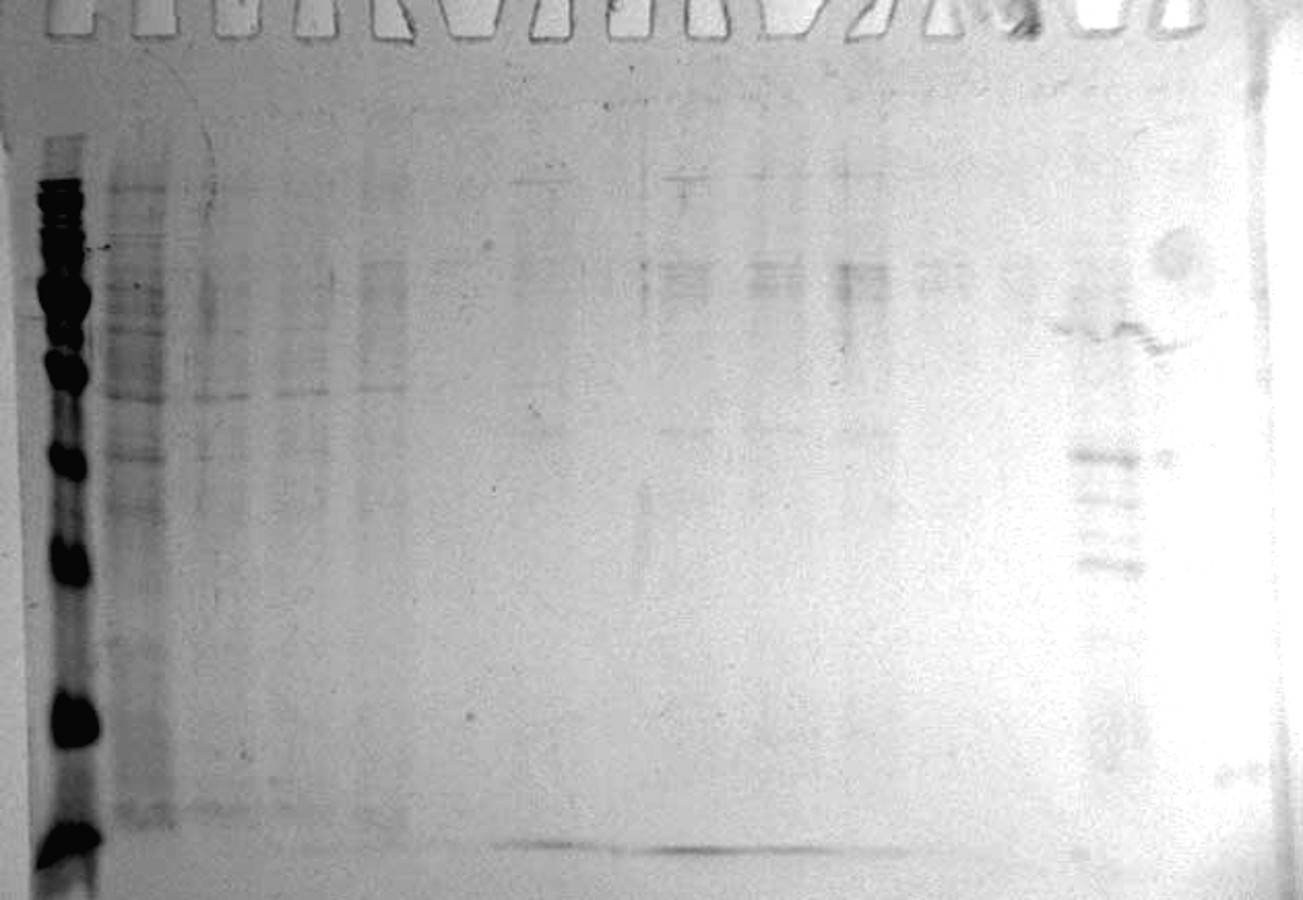

Supplement: Supplemental Information 4 — Lanes: M, molecular weight markers; 1, unbound proteins fraction; 2–3, fractions eluted in 10 mM NaCl; 4, fraction eluted in 30 mM NaCl; 5–7, fractions eluted in 50 mM NaCl: 8–10, fractions eluted in 100 mM NaCl; 11–12, fractions eluted in 500 mM NaCl; 13, fraction eluted in 1.6 M NaCl. [file peerj-13-19989-s004.jpg]
